# A novel doxorubicin-glucuronide prodrug DOX-GA3 for tumour-selective chemotherapy: distribution and efficacy in experimental human ovarian cancer

**DOI:** 10.1054/bjoc.2000.1640

**Published:** 2001-02

**Authors:** P H J Houba, E Boven, I H van der Meulen-Muileman, R G G Leenders, J W Scheeren, H M Pinedo, H J Haisma

**Affiliations:** Department of Medical Oncology, University Hospital Vrije Universiteit, P.O. Box 7057, Amsterdam, MB, 1007, The Netherlands; Department of Organic Chemistry, University of Nijmegen, P.O. Box 9010, Nijmegen, GL, 6500, The Netherlands

**Keywords:** anthracyclines, cancer chemotherapy, β-glucuronidase, glucuronide

## Abstract

The doxorubicin (DOX) prodrug *N* -[4-doxorubicin- *N* -carbonyl (oxymethyl) phenyl] *O* -β-glucuronyl carbamate (DOX-GA3) was synthesised for specific activation by human β-glucuronidase, which is released in necrotic areas of tumour lesions. This novel prodrug was completely activated to the parent drug by human β-glucuronidase with V _max_= 25.0 μmol min^–1^mg^–1^and K _m_= 1100 μM. The pharmacokinetics and distribution of DOX-GA3 in nude mice bearing human ovarian cancer xenografts (OVCAR-3) were determined and compared with DOX. Administration of DOX at 8 mg kg^–1^i.v. (maximum tolerated dose, MTD) to OVCAR-3-bearing mice resulted in a peak plasma concentration of the drug of 16.4 μM (*t* = 1 min). A 7.6-times lower peak plasma concentration of DOX was measured after injection of DOX-GA3 at 250 mg kg^–1^i.v. (50% of MTD). In normal tissues the prodrug showed peak DOX concentrations that were up to 5-fold (heart) lower than those found after DOX administration. DOX-GA3 activation by β-glucuronidase in the tumour yielded an almost 5-fold higher DOX peak concentration of 9.57 nmol g^–1^(*P*< 0.05) than the peak concentration of only 2.14 nmol g^–1^observed after DOX. As a consequence, the area under the curve of DOX calculated in tumour tissue after DOX-GA3 (13.1 μmol min^–1^g^–1^) was 10-fold higher than after DOX (1.31 μmol min^–1^g^–1^). The anti-tumour effects of DOX-GA3 and DOX were compared at equitoxic doses in OVCAR-3 xenografts at a mean tumour size of 125 mm^3^. The prodrug given i.v. at 500 mg kg^–1^weekly × 2 resulted in a maximum tumour growth inhibition of 87%, while the standard treatment with DOX at a dose of 8 mg kg^–1^i.v. weekly × 2 resulted in a maximum tumour growth inhibition of only 56%. Treatment with DOX-GA3 was also given to mice with larger tumours containing more necrosis. For tumours with a mean size of 400 mm^3^the specific growth delay by DOX-GA3 increased from 2.7 to 3.9. Our data indicate that DOX-GA3 is more effective than DOX and suggest that the prodrug will be specifically advantageous for treatment of advanced disease. © 2001 Cancer Research Campaign http://www.bjcancer.com


*British Journal of Cancer* (2001) **84**(4), 550–557
© 2001 Cancer Research Campaign
doi: 10.1054/ bjoc.2000.1640, available online at http://www.idealibrary.com on IDEAL®

http://www.bjcancer.com

### A novel doxorubicin-glucuronide prodrug DOX-GA3 for tumour-selective chemotherapy: distribution and efficacy in experimental human ovarian cancer

**PHJ Houba[1], E Boven[1], IH van der Meulen-Muileman[1], RGG Leenders[2], JW Scheeren[2], HM Pinedo[1] and HJ Haisma[1]**

[1]Department of Medical Oncology, University Hospital Vrije Universiteit, P.O. Box 7057, 1007 MB Amsterdam, The Netherlands; [2]Department of Organic Chemistry, University of Nijmegen, P.O. Box 9010, 6500 GL Nijmegen, The Netherlands

**Summary** The doxorubicin (DOX) prodrug *N*-[4-doxorubicin-*N*-carbonyl (oxymethyl) phenyl] *O*-β-glucuronyl carbamate (DOX-GA3) was synthesised for specific activation by human β-glucuronidase, which is released in necrotic areas of tumour lesions. This novel prodrug was completely activated to the parent drug by human β-glucuronidase with $V_{max}$ = 25.0 μmol min$^{-1}$mg$^{-1}$ and $K_m$ = 1100 μM. The pharmacokinetics and distribution of DOX-GA3 in nude mice bearing human ovarian cancer xenografts (OVCAR-3) were determined and compared with DOX. Administration of DOX at 8 mg kg$^{-1}$ i.v. (maximum tolerated dose, MTD) to OVCAR-3-bearing mice resulted in a peak plasma concentration of the drug of 16.4 μM ($t$ = 1 min). A 7.6-times lower peak plasma concentration of DOX was measured after injection of DOX-GA3 at 250 mg kg$^{-1}$ i.v. (50% of MTD). In normal tissues the prodrug showed peak DOX concentrations that were up to 5-fold (heart) lower than those found after DOX administration. DOX-GA3 activation by β-glucuronidase in the tumour yielded an almost 5-fold higher DOX peak concentration of 9.57 nmol g$^{-1}$ ($P$ < 0.05) than the peak concentration of only 2.14 nmol g$^{-1}$ observed after DOX. As a consequence, the area under the curve of DOX calculated in tumour tissue after DOX-GA3 (13.1 μmol min$^{-1}$ g$^{-1}$) was 10-fold higher than after DOX (1.31 μmol min$^{-1}$ g$^{-1}$). The anti-tumour effects of DOX-GA3 and DOX were compared at equitoxic doses in OVCAR-3 xenografts at a mean tumour size of 125 mm$^3$. The prodrug given i.v. at 500 mg kg$^{-1}$ weekly × 2 resulted in a maximum tumour growth inhibition of 87%, while the standard treatment with DOX at a dose of 8 mg kg$^{-1}$ i.v. weekly × 2 resulted in a maximum tumour growth inhibition of only 56%. Treatment with DOX-GA3 was also given to mice with larger tumours containing more necrosis. For tumours with a mean size of 400 mm$^3$ the specific growth delay by DOX-GA3 increased from 2.7 to 3.9. Our data indicate that DOX-GA3 is more effective than DOX and suggest that the prodrug will be specifically advantageous for treatment of advanced disease. © 2001 Cancer Research Campaign http://www.bjcancer.com

**Keywords**: anthracyclines; cancer chemotherapy; β-glucuronidase; glucuronide

Doxorubicin (DOX) is an anticancer agent with a wide spectrum of activity. Cumulative dose-related cardiotoxicity, however, is a major side-effect of DOX, in addition to the acute toxicities, such as myelosuppression, nausea and vomiting. The success of DOX, and its limitations in clinical use, have directed research endeavours for the development of analogues of DOX with an improved therapeutic index. Among these are iododoxorubicin, AD-32 and epidoxorubicin (Weiss, 1992). Iododoxorubicin was noted to be promising in phase I trials, but in phase II trials the response rate was too low to warrant further development. AD-32 has greater anti-tumour activity, and less cardiotoxicity, than DOX, but drug formulation and solubility problems prevented its further clinical development. Of the available analogues, only epidoxorubicin appears to have a reduced cardiotoxicity with retention of anti-tumour activity and is in use in current cancer chemotherapy.

Another way to improve the selectivity and efficacy of chemotherapy is the use of non-toxic prodrugs that are preferentially converted into active anticancer agents at the tumour site (Sinhababu and Thakker, 1996). *N*-L-leucyl-DOX is a prodrug of DOX, to be activated by tumour peptidases (Deprez-de Campeneere et al, 1982). In human ovarian cancer xenografts, *N*-L-leucyl-DOX was more effective than DOX (Boven et al, 1992). Clinical studies on *N*-L-leucyl-DOX, however, have indicated premature activation of the prodrug in the circulation, because of which the selectivity of the prodrug would be reduced (de Jong et al, 1992). Elevated enzyme levels in tumour tissue have been reported for β-glucuronidase (Connors and Whisson, 1966). Bosslet et al (1995) and Schumacher et al (1996) have shown that this enzyme is released in the extracellular space as a result of necrosis in tumours. The enzyme can only be detected in very low concentrations in the circulation (Fishman, 1970). Therefore, β-glucuronidase may be exploited for the specific activation of glucuronide prodrugs in tumour tissue.

We have developed glucuronide derivatives of anthracyclines and showed that these prodrugs, such as epirubicin-glucuronide (Haisma et al, 1992) and daunorubicin-glucuronides (Leenders et al, 1995; Houba et al, 1996), are relatively non-toxic in vitro and can be activated by β-glucuronidase to yield the active anthracycline. Treatment with the glucuronide prodrug daunorubicin-GA3 (DNR-GA3) induced a better tumour growth delay than daunorubicin (DNR) when studied at equitoxic doses in 3 human ovarian cancer xenografts which were sensitive to DNR (Houba et al, 1998). Comparison of the distribution and pharmacokinetics of DNR and DNR-GA3 demonstrated that the prodrug DNR-GA3 was selectively activated by human β-glucuronidase present in the tumours and resulted in a higher DNR AUC in tumours and lower

*Received 5 July 2000*
*Revised 7 November 2000*
*Accepted 17 November 2000*

*Correspondence to:* HJ Haisma. Present address: Dept of Therapeutic Gene Modulation, University Centre for Pharmacy, University of Groningen, PO Box 196, 9700 AD Groningen, The Netherlands. E-mail: h.j.haisma@farm.rug.nl

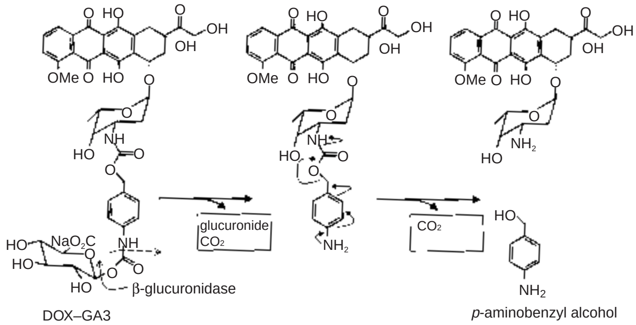

**Figure 1** Chemical structure of prodrug *N*-[4-doxorubicin-*N*-carbonyl (oxymethyl) phenyl] *O*-β-glucuronyl carbamate (DOX-GA3). After hydrolysis DOX-GA3 is activated to DOX; glucuronic acid and 4-aminobenzyl alcohol (spacer) are released

DNR AUCs in normal tissues (Houba et al, 1999). Our treatment results with a DNR prodrug are encouraging, but solid tumour types show better sensitivity to DOX than to DNR. In analogy, this has led to the synthesis of the prodrug of DOX: *N*-[4-doxorubicin-*N*-carbonyl (oxymethyl) phenyl] *O*-β-glucuronyl carbamate (DOX-GA3; Figure 1). DOX-GA3 is a derivative of DOX in which the glucuronic acid moiety is linked to the anthracycline via a synthetic spacer, designed to increase the hydrolysis by human β-glucuronidase. We anticipate that this prodrug may have a broader application in the treatment of cancer.

In the present experiments we compared the antiproliferative effects of DOX-GA3 and DOX in vitro, and calculated the $K_m$ and $V_{max}$ of DOX-GA3 hydrolysed by human β-glucuronidase. We studied whether DOX-GA3 was specifically activated to DOX by β-glucuronidase in tumour tissue. To this end, the distribution of DOX released from DOX-GA3 was compared with that of DOX alone in mice bearing OVCAR-3 xenografts in tumour tissue as well as in normal organs at several time-points after injection. After determination of the maximum tolerated dose (MTD) of DOX-GA3 in OVCAR-3-bearing nude mice, we compared the anti-tumour efficacy of DOX-GA3 with that of DOX. As large tumours have more necrosis than small tumours and are therefore expected to contain higher levels of extracellular β-glucuronidase, special attention was paid to the influence of the tumour size on drug effects.

### MATERIALS AND METHODS

### Materials

The human ovarian cancer cell line NIH:OVCAR-3 (Hamilton et al, 1983) was grown in Dulbecco's modified Eagle's medium (Life Technologies, Paisley, Scotland) supplemented with 10% heat- inactivated fetal calf serum (ICN, Costa Mesa, CA), 50 IU $ml^{-1}$ penicillin (ICN) and 50 μg $ml^{-1}$ streptomycin (ICN) in a humidified atmosphere containing 5% $CO_2$ at 37˚C.

Doxorubicin (DOX, Pharmachemie BV, Haarlem, The Netherlands) was purchased as a powder. The prodrug *N*-[4-doxorubicin-*N*-carbonyl (oxymethyl) phenyl] *O*-β-glucuronyl carbamate (DOX-GA3) was synthesized as described (Leenders et al, 1999). Stock solutions of DOX and DOX-GA3 were prepared in sterile water and stored at –20˚C.

### Characterization of DOX-GA3 in vitro

The purity and stability of DOX-GA3 and the activation of DOX-GA3 in the presence of β-glucuronidase was determined in 0.1% (w/v) BSA/PBS at pH 6.8 and 37˚C. Samples were taken after 24 h and were analysed with reversed-phase HPLC as described (Houba et al, 1999).

In each cluster of runs standards of DOX-GA3 and DOX were included, at the beginning and at the end of the cluster. The peak areas from the elution peaks in the chromatograms were determined by integration using the program Gyncosoft (Gynkotek, Version 5.3E). Calibration of the system was performed as described (de Jong et al, 1991) and the detection limit was 0.01 μM (pro)drug.

The half-life time of hydrolysis of the prodrug by human recombinant β-glucuronidase (Houba et al, 1996) was determined in PBS at 37˚C and pH 6.8, reflecting the tumour interstitial pH (Martin and Jain, 1994), at concentrations of the enzyme (1 μg $ml^{-1}$) and the prodrug (100 μM) assumed to be clinically relevant (Houba et al, 1999). The prodrug and the enzyme were diluted in 0.1% (w/v) BSA/PBS at pH 6.8. After incubation at 37˚C the reaction was stopped by adding methanol. Samples were analysed by HPLC as described above.

To further characterize the enzymatic conversion of the prodrugs by human β-glucuronidase $K_m$ and $V_{max}$ values were determined. A range of prodrug concentrations ($5 \times 10^{-5}$ to $6 \times 10^{-3}$ M) was tested against a human β-glucuronidase concentration of 1 μg $ml^{-1}$ to determine the hydrolysis expressed as $K_m$ and $V_{max}$ values. After incubation for 30 min at 37˚C the reaction was stopped by adding methanol. Samples were analysed by HPLC and $K_m$ and $V_{max}$ values were calculated from direct linear plots as described by Eisenthal and Cornish-Bowden (Eisenthal and Cornish-Bowden, 1974).

### Antiproliferative effects

The in vitro antiproliferative effects of drug and prodrug were determined in OVCAR-3 cells as previously described (Houba et al, 1996). The antiproliferative effect was expressed as the $IG_{50}$ value, which is the (pro)drug concentration that gives 50% growth inhibition when compared with control cell growth.

### Human ovarian cancer xenograft OVCAR-3

All animal experiments were approved by the Institution Animal Experiments Committee and performed in accord with the institution guidelines. Female athymic nude mice (Hsd: athymic nude-*nu*; Harlan Cpb, Horst, The Netherlands) were handled under specified pathogen-free conditions. The human ovarian cancer xenograft OVCAR-3 has been described earlier (Molthoff et al, 1991). The histology shows a poorly differentiated serous adenocarcinoma and tumours have a mean volume-doubling time of 5.0 days. Tumours from previous recipients were transferred by implanting tissue fragments with a diameter of 2–3 mm into both flanks of 8–10-week-old mice. Upon growth, tumours were measured twice a week by the same observer. The tumour volume was calculated by the equation length × width × thickness × 0.5, and expressed in $mm^3$.

### Maximum tolerated dose of DOX and DOX-GA3

The MTD was defined as the dose that gives a mean reversible loss of approximately 10% of the initial weight within 2 weeks after the first injection. Animals dying within 2 weeks after the final injection were considered as toxic deaths. The MTD of DOX in

© 2001 Cancer Research Campaign 

OVCAR-3-bearing mice was established earlier being 8 mg $kg^{-1}$ i.v. weekly × 2 (Boven et al, 1990). The MTD of DOX-GA3 in OVCAR-3-bearing mice was found to be 500 mg $kg^{-1}$ i.v. weekly × 2. No toxic deaths occurred and the maximum weight loss was approximately 10%.

### Distribution of DOX and DOX-GA3 in tumour and normal tissues

Nude mice bearing s.c. xenografts with a mean volume of 600 $mm^3$ were injected i.v. with DOX (8 mg $kg^{-1}$) or DOX-GA3 (250 mg $kg^{-1}$) to determine their distribution and pharmacokinetics. Because of the limited availability of DOX-GA3 we investigated DOX-GA3 at half of the MTD. At different time-points ranging from 1 min to 24 h blood, tumours, liver, heart and kidneys were removed in groups of three OVCAR-3-bearing mice per time-point. Blood was collected in heparinized vials and centrifuged at 16 000 ***g*** for 5 min. To plasma 1/100 volume of 100 mM D-glucaric acid-1,4-lactone monohydrate (saccharolactone; Fluka, Buchs, Germany) was added to prevent hydrolysis of DOX-GA3 by β-glucuronidase. Saccharolactone is a selective competitive inhibitor of activity of β-glucuronidase. The activity of the enzyme was almost completely abolished (>99%) at the used concentration of 1 mM (data not shown).

After blood sampling the tumour, liver, heart and kidney tissues were collected and immediately frozen in liquid nitrogen and stored at –20˚C until preparation for HPLC analysis (Houba et al, 1999). In control tissue homogenates and serum samples, incubated with DOX-GA3 at concentrations up to 1 mM and stored for up to 4 weeks at –20˚C, less than 0.1% activation to doxorubicin was observed (data not shown).

Data were analysed with the software program Topfit, in order to calculate half-life times and area under the concentration versus time curves (AUC). Differences between drug concentrations per time-point were evaluated with Student's *t*-test.

### Drug targeting index (DTI)

The efficiency of targeting can be described as a drug targeting index (Kearney, 1996). Assuming that the AUCs at the response (tumour) and toxic sites (bone marrow, heart) reflect the relative activity and toxicity of the drug, the DTI can be defined as follows:

$$\mathrm{DTI} = \frac{\mathrm{AUC\ (R,\ target)\ /\ AUC\ (T,\ target)}}{\mathrm{AUC\ (R,\ non\text{-}target)\ /\ AUC\ (T,\ non\text{-}target)}}$$

where R represents the response site (tumour) and T the toxic site after targeted delivery (DOX-GA3, target) and non-targeted delivery (DOX, non-target), respectively. A value higher than 1 indicates targeting to the response site.

### Anti-tumour activity of anthracyclines in vivo

Mice bearing OVCAR-3 tumours were used to compare DOX with DOX-GA3 anti-tumour effects. At the start of the treatment (day 0), mice were grouped in order to obtain similarities in the mean tumour volume. For small tumours the mean volume was 125 $mm^3$ and for larger tumours 400 $mm^3$. Control and treatment groups consisted of 6 animals each. DOX was given in a dose of 8 mg $kg^{-1}$ i.v. weekly × 2 (MTD) to mice with small and larger tumours. DOX-GA3 was studied at 500 mg $kg^{-1}$ i.v. weekly × 2 (MTD) in groups with small and larger tumours. Mice were weighed twice a week and tumours were measured on the same days.

For evaluation of drug effects the relative tumour volume was expressed by the formula $V_T/V_0$, where $V_T$ is the volume on any given day and $V_0$ is the volume on day 0. The ratio between the mean of the relative volumes of treated tumours and that of control tumours × 100% (T/C%) was assessed on each day of measure-ment and used to calculate the percentage of maximum GI (GI = 100%– T/C%). The relative tumour volume was calculated and normalized for growth of the control tumours. As a measure of the duration of the treatment effects, the days for each tumour to double twice in volume ($T_{D1\to4}$) was calculated. If a tumour did not reach 2 volume doubling times this volume was extrapolated from the last 2 available measurements. Differences in mean $T_{D1\to4}$ between groups were evaluated with Student's *t*-test. In addition, differences in efficacy between the treatment groups of small vs large tumours were expressed as the specific growth delay (SGD) (Boven et al, 1988). The SGD was calculated according to the following formula:

$$\mathrm{SGD} = \frac{T_{D1\to4}\ \mathrm{treated} - T_{D1\to4}\ \mathrm{control}}{T_{D1\to4}\ \mathrm{control}}$$

### RESULTS

### Characterization of DOX-GA3 in vitro

Our initial experiments sought to determine enzymatic activation of the glucuronide prodrug DOX-GA3 to the parent drug DOX. To this end DOX-GA3 was incubated with human β-glucuronidase. HPLC analysis showed that DOX-GA3 was more than 99.9% pure and could be completely hydrolysed to DOX in the presence of excess human β-glucuronidase to DOX, and no spacer-drug intermediate was detectable. A sample chromatogram from an intermediate timepoint (30 min) is shown in Figure 2. The prodrug, incubated at the clinically relevant concentrations of 100 μM for DOX-GA3 and of 1 μg $ml^{-1}$ for the enzyme, was hydrolysed at pH 6.8 and 37˚C with a $t^1/_2$ of 140 min.

Based on the in vitro cleavage experiments at different prodrug concentrations at pH 6.8 and at 37˚C, $V_{max}$ and $K_m$ were calculated from direct linear plots as described by Eisenthal (Eisenthal and Cornish-Bowden, 1974). For DOX-GA3 a $V_{max}$ of 25.0 μmol $min^{-1}$ $mg^{-1}$ and a $K_m$ of 1100 μM was found.

We next analysed the in vitro antiproliferative effects of DOX and DOX-GA3. For DOX the $IC_{50}$ was 0.12 μM (SD ± 0.06) and the prodrug was more than 100-times less capable of inhibiting cell growth with an $IC_{50}$ value of 22 μM (SD ± 7.6). This was probably caused by activation of the prodrug through β-glucuronidase released from dead cells because we were unable to detect DOX in the prodrug preparation. When DOX-GA3 was incubated with an excess of β-glucuronidase, the antiproliferative effect returned to a value similar to that of DOX (0.16 μM; SD ± 0.06). This indicated that the prodrug was completely activated to the parent drug under these conditions (Figure 3).

### Distribution and kinetics in plasma, tumour and normal tissues

The distribution of DOX (8 mg $kg^{-1}$) and DOX-GA3 (250 mg $kg^{-1}$) in plasma, tumour and normal tissues was compared in

 © 2001 Cancer Research Campaign

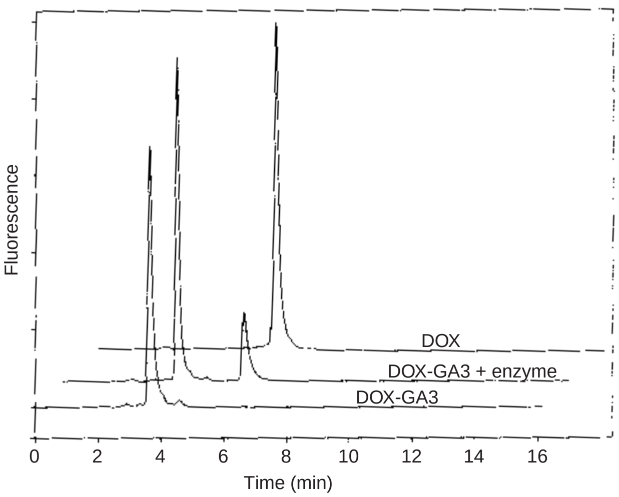

**Figure 2** HPLC chromatograms of DOX (100 μM, upper line), DOX-GA3 (100 μM, lower line), and DOX-GA3 (100 μM, middle line) after partial hydrolysis by human β-glucuronidase at 1 μg $ml^{-1}$ for 30 min at pH 6.8 and 37˚C. The chromatographic conditions are given in the Materials and Methods section. For clarity, the chromatograms have been offset 1 min in the time axis

OVCAR-3-bearing mice (Figure 4). First, the in vivo stability of the prodrug was tested in non-tumour bearing mice. Administration of DOX-GA3 (250 mg $kg^{-1}$) in these animals resulted in a drug/prodrug ratio of 0.001 ($t$ = 30 min) in plasma and no other major peaks were visible. These findings indicate the lack of activation by endogenous β-glucuronidase in non-tumour-bearing animals. Next, we investigated this effect in tumour-bearing animals. After administration of DOX-GA3, the prodrug peak concentration was 1370 nmol $ml^{-1}$ ($t$ =1 min) in plasma and a drug peak concentration of 2.17 nmol $ml^{-1}$ was found. After administration of DOX, a considerably higher peak concentration of 16.4 nmol $ml^{-1}$ was detected in plasma. The ratio of drug/prodrug (0.002) was similar to that found in non-tumour-bearing animals, indicating that the systemic activation of prodrug is not increased by the presence of tumour.

DOX administration resulted in DOX peak concentrations in mouse organs that where highest in kidney (90.2 nmol $g^{-1}$) followed by liver (46.0 nmol $g^{-1}$), and heart (15.9 nmol $g^{-1}$). Prodrug injection also resulted in highest peak DOX concentrations in kidney (70.8 nmol $g^{-1}$) followed by liver (14.2 nmol $g^{-1}$) and heart (3.27 nmol $g^{-1}$) at 30 min to 2 h after injection of the prodrug. These values were 2 to 5 times lower than those found after DOX administration, except for kidney tissue where it was slightly lower (Figure 4).

Administration of DOX resulted in a peak concentration of the drug in OVCAR-3 tumour tissue of 2.14 nmol $g^{-1}$ at 1 min after injection (Figure 4). The prodrug DOX-GA3 had a peak of DOX-GA3 of 44.0 nmol $g^{-1}$ ($t$ = 30 min). A significantly higher DOX level of 9.57 nmol $g^{-1}$ ($t$ = 2 h) was found after prodrug injection ($P < 0.001$; $t$ = 2 h) than after administration of the parent drug. In tumours, DOX was well retained both after DOX and DOX-GA3 administration, and the elimination of DOX from the tumour was not different after drug or after prodrug administration (Figure 4).

As an example for the selective activation of the prodrug at the tumour site, the ratios of the DOX concentrations at 2 h after DOX-GA3 and DOX administration are given for plasma, normal tissues and tumour tissue in Figure 5. At this time, the highest DOX level in tumour tissue was measured after prodrug administration. It is clear that the DOX concentrations in normal tissues were lower after prodrug than after DOX administration, except for the kidney.

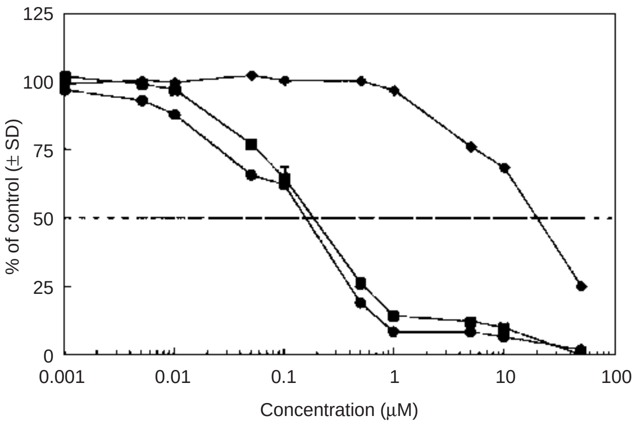

**Figure 3** In vitro antiproliferative effects in OVCAR-3 cells exposed to various concentrations of DOX or DOX-GA3. Cell growth was measured after 72 h by sulforhodamine B staining and was expressed as the percentage of growth relative to control cells. (●) DOX; (◆) DOX-GA3; (■) DOX-GA3 in the presence of an excess of human β-glucuronidase. Bars, ± SD

### Areas under the concentration versus time curve

As a measure of drug exposure, the AUC of DOX was calculated for plasma, normal mouse organs and tumour tissue by the trapezoidal rule using the available time-points and the software program Topfit (Table 1). The plasma AUC of DOX after administration of DOX (0.342 μmol min $ml^{-1}$) was 8-fold higher than the AUC of DOX after injection of DOX-GA3 (0.042 μmol $min^{-1}$ $ml^{-1}$), with DOX given at the MTD and prodrug at half of the MTD. For the liver and the heart the respective AUCs for DOX were 1.8- and 3.1-fold higher and for the kidney the AUC was 2.0 times lower after DOX than after prodrug administration. Of importance, in OVCAR-3 tumour tissue the AUC of DOX from DOX-GA3 was 10-fold higher than that after DOX.

### Drug targeting index (DTI)

The efficiency of targeting can be described as a drug targeting index (Kearney, 1996). We calculated the DTI for plasma (as a surrogate for bone marrow) and heart tissue because these are the major organs for anthracycline toxicity. For plasma a high DTI of 81.4 was calculated and for heart tissue the DTI was 31.4. This analysis thus confirms the selective activation of the prodrug DOX-GA3 in tumours.

### Anti-tumour activity of DOX and DOX-GA3 in vivo

The administration of DOX (8 mg $kg^{-1} \times 2$) resulted in a maximum $G_1$ of 56% in small OVCAR-3 xenografts. At an equitoxic dose the molar amount of DOX-GA3 (500 mg $kg^{-1} \times 2$) that could be administered was 38-fold higher when compared with that of the parent drug. In OVCAR-3 xenografts DOX-GA3 induced a maximum $G_1$ of 87%, which was considerably higher than that of DOX (Table 2, Figure 6). The augmented anti-tumour effect of

© 2001 Cancer Research Campaign

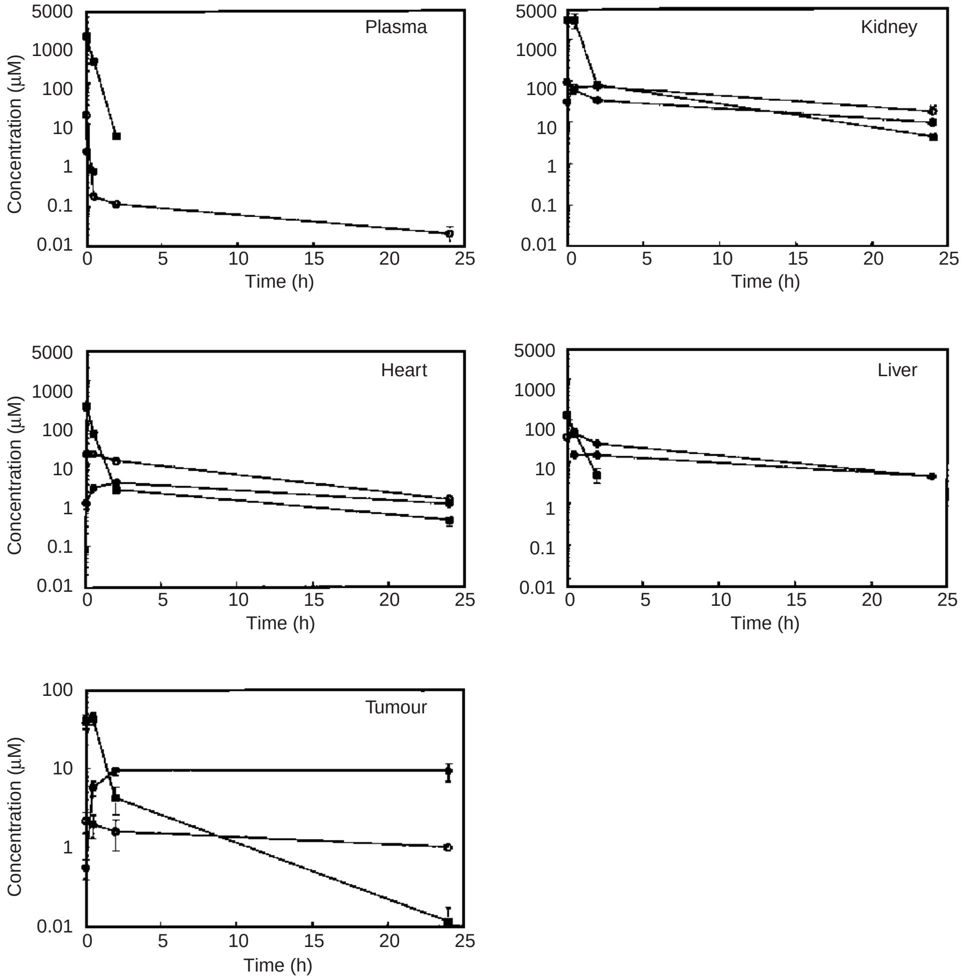

**Figure 4** The concentration–time curves of: DOX (○) as a result of the administration of 8 mg $kg^{-1}$ DOX; DOX (●) as a result of the administration of 250 mg $kg^{-1}$ DOX-GA3; and DOX-GA3 (■) after 250 mg $kg^{-1}$ DOX GA3, in plasma, tumours, liver, heart and kidneys of OVCAR-3-bearing mice. Bars, ± SD

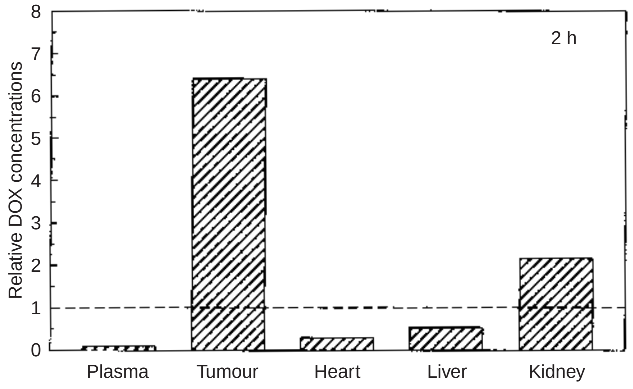

**Figure 5** Ratio in mouse plasma, normal organs and OVCAR-3 tumour tissue between DOX concentrations obtained after DOX-GA3 or DOX administration. The concentrations of DOX (µM) in tissues 2 h after injection of DOX-GA3 at 250 mg $kg^{-1}$ or DOX at 8 mg $kg^{-1}$ were measured by reversed-phase HPLC. The ratio was calculated by dividing the DOX concentration obtained after DOX-GA3 administration, by the DOX concentration after DOX administration. For all tissues the DOX concentrations were significantly different for drug or prodrug administration ($P < 0.05$, Student's *t*-test)

DOX-GA3 was also demonstrated by an increase of the duration of the anti-tumour effect, expressed as $T_{D1\rightarrow4,}$ from 11.5 to 32.4 days, which was significant ($P < 0.01$, Table 2).

To study the effect of tumour size on the tumour growth inhibition DOX-GA3 was administered to animals bearing small or larger tumours, where larger tumours are expected to have more necrosis and, thus, more β-glucuronidase in the extracellular space. Larger tumours grew much slower than small tumours ($T_{D1\rightarrow4,}$ small 8.0 days, large 15.4 days). In animals treated with DOX, the maximum growth inhibition was similar for both small and larger tumours, 56 and 52% respectively. DOX-GA3 treatment of larger tumours indeed resulted in a better inhibition of growth. The SGD in OVCAR-3 tumours increased from 2.7 to 3.9 for small and larger tumours, respectively.

### DISCUSSION

In this study we describe a new doxorubicin prodrug, DOX-GA3, designed for specific activation to DOX at the tumour site. In nude mice bearing human ovarian cancer xenografts we showed that equitoxic doses of (pro)drug resulted in better anti-tumour

 © 2001 Cancer Research Campaign

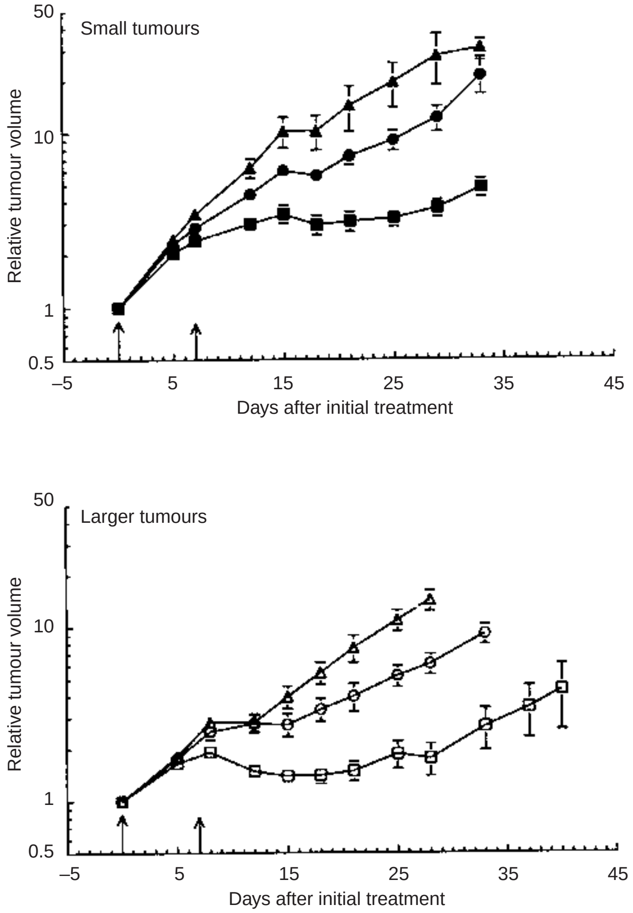

**Figure 6** Tumour growth in mice bearing small or larger OVCAR-3 xenografts after i.v. treatment with maximum tolerated doses of DOX (day 0, 7) or DOX-GA3 (day 0,7). ▲, control (small tumours); ●, DOX 8 mg kg$^{-1}$ (small tumours); ■, DOX-GA3 500 mg kg$^{-1}$ (small tumours); △, control (larger tumours); ○, DOX 8 mg kg$^{-1}$ (larger tumours); □, DOX-GA3 500 mg kg$^{-1}$ (larger tumours). Bars, ± SEM

effects after DOX-GA3 than after DOX. The administration of DOX-GA3 led to specific activation of the prodrug at the tumour site yielding higher concentrations of DOX in tumour tissue than after DOX injection. The 10-fold higher AUC of DOX from DOX-GA3 thus explains the better anti-tumour effect measured for DOX-GA3 when compared with DOX. Furthermore, the efficacy of DOX-GA3 appeared to be more pronounced in larger tumours when compared with the efficacy in small tumours. In the animals treated with DOX, the larger tumours were not more sensitive than the smaller tumours.

Our findings are consistent with other studies employing this approach. In this regard, as early as 1966 Connors and Whisson (Connors and Whisson, 1966) have shown that glucuronide prodrugs can be activated in tumours by β-glucuronidase. β-Glucuronidase is present extracellularly in necrotic tumour areas found in tumours larger than 3 mm diameter (Bosslet et al, 1995; Schumacher et al, 1996), whereas in normal tissues it is present only intracellularly inside lysosomes and microsomes (Levvy and Conchie, 1966). A clinical trial of aniline mustard was performed in patients with advanced cancer (Young et al, 1976). The drug used, aniline mustard, is transformed into the prodrug p-hydroxy aniline glucuronide which acts as a substrate for β-glucuronidase found in tumours. A correlation was shown between β-glucuronidase activity and patient response. However, the necessary high levels of β-glucuronidase were observed only rarely. The limited success of this trial is possibly due to the slow activation of the aniline glucuronide by the enzyme. The glucuronide prodrugs DNR-GA3 and DOX-GA3 were designed to be rapidly activated by human β-glucuronidase at a pH of 6.8 (Houba et al, 1996).

For DOX-GA3 a $V_{max}$ of 25.0 μmol min$^{-1}$ mg$^{-1}$ and a $K_m$ of 1100 μM was found. The relatively high $K_m$ is slightly lower than the $K_m$ observed with the synthetic substrate substrate 4-nitrophenyl-glucuronide (Tomino and Paigen, 1975) (1.3 mM) and another glucuronide prodrug (1.3 mM) described by Bosslet et al (1994). The relatively high $K_m$ may be beneficial for improving tumour:non-tumour ratios of active drug: prodrug activation will

**Table 1** AUC[a] of DOX in plasma and tissues of OVCAR-3-bearing mice after i.v. administration of DOX (8 mg kg$^{-1}$) or DOX-GA3 (250 mg kg$^{-1}$)

| Treatment | Plasma (μmol min$^{-1}$ ml$^{-1}$) | Liver (μmol min$^{-1}$ g$^{-1}$) | Heart (μmol min$^{-1}$ g$^{-1}$) | Kidney (μmol min$^{-1}$ g$^{-1}$) | Tumour (μmol min$^{-1}$ g$^{-1}$) |
|---|---|---|---|---|---|
| DOX | 0.342 | 24.5 | 9.70 | 33.2 | 1.31 |
| DOX-GA3 | 0.042 | 13.4 | 3.09 | 65.0 | 13.1 |

[a]The AUC (0–24 h) using the available time-points.

**Table 2** Anti-tumour effect with DOX or DOX-GA3 in mice bearing OVCAR-3 xenografts

| Treatment | Size | Dose i.v. (mg kg$^{-1}$) | Days | Tumour volume[a] mean ± SEM | Weight loss % ± SD | Weight day 14 % ± SD | GI %[c] | $T_{D1\rightarrow4}$[b] days ± SEM (*n*) |
|---|---|---|---|---|---|---|---|---|
| Control | Small | | | 128 ± 29 | n.a. | 111.0 ± 4.7 | n.a. | 8.0 ± 0.6 (10) |
| DOX | Small | 8 | 0,7 | 123 ± 19 | 1.3 ± 2.0 | 104.4 ± 3.4 | 56 | 11.5 ± 1.8 (10) |
| DOX-GA3 | Small | 500 | 0,7 | 123 ± 19 | 5.1 ± 3.4 | 99.0 ± 3.1 | 87 | 32.4 ± 5.4 (11)[d,e] |
| Control | Large | | 0,7 | 390 ± 97 | n.a. | 106.8 ± 5.5 | n.a. | 15.4 ± 1.4 (10) |
| DOX | Large | 8 | 0,7 | 534 ± 128 | 2.8 ± 0.2 | 101.5 ± 2.8 | 52 | 21.5 ± 0.7 (5) |
| DOX-GA3 | Large | 500 | 0,7 | 458 ± 107 | 9.6 ± 7.2 | 91.9 ± 6.5 | 88 | 75.1 ± 11.8 (10)[d] |

n.a., not applicable; [a]at start of treatment; [b]tumour volume doubling time in days from a relative volume of 1 to 4; [c]maximum growth inhibition; [d]*P* < 0.001 when compared with control; [e]*P* < 0.01 when compared with DOX.

© 2001 Cancer Research Campaign 

be relatively slow at sites with low levels of enzyme. In this regard, a $K_m$ of 1 mM was shown to be optimal by pharmacokinetic modelling by Yuan et al (1991). The half-life of hydrolysis at clinically relevant concentrations of the prodrug and human β-glucuronidase was 140 min. Although this seems to be far from optimal, the time to reach toxic concentrations of DOX in tumour tissue is much shorter, only a few minutes (Figure 4).

DOX is a lipophilic molecule and, as a consequence, it rapidly penetrates into tissues. Therefore, normal tissue DOX levels are relatively high after DOX administration and may account for unfavourable side-effects (Figure 4). The hydrophilic glucuronide moiety of DOX-GA3 prevents rapid diffusion of prodrug into cells. This is confirmed by the high peak plasma concentrations of DOX-GA3 and its rapid clearance from plasma, tumour and normal tissues.

In plasma, heart and liver tissues the concentrations of DOX were lower after DOX-GA3 administration than after injection of DOX itself. Lower concentrations of DOX in the heart are of advantage, because this organ is the site of cumulative dose-limiting toxicity of DOX. In the kidney, higher concentrations of DOX from DOX-GA3 than after DOX were measured. These higher concentrations may be explained by the presence of β-glucuronidase in the renal tubular cells. This should not result in increased toxicity as in patients renal insufficiency is rarely observed after DOX administration.

In OVCAR-3 xenografts higher concentrations of DOX from DOX-GA3 were detectable than after administration of DOX. Presumably, DOX-GA3 was activated by β-glucuronidase present in tumour tissue in the extracellular space, but not in normal tissues. Therefore, higher AUC values of DOX from DOX-GA3 were reached than after DOX administration. These higher AUC values explain the better inhibition of tumour growth in OVCAR-3 xenografts observed after DOX-GA3 administration when compared with DOX, when given at an equitoxic dose. The $T_{D1\rightarrow4}$ increased significantly ($P < 0.001$) from 11.5 days (8 mg $kg^{-1}$ DOX weekly × 2) to 32.4 days (500 mg $kg^{-1}$ DOX-GA3 weekly × 2).

It was hypothesized that larger tumours contain more necrosis and, thus, more β-glucuronidase would be available to activate DOX-GA3, as has also been described by the group of Bosslet (Bosslet et al, 1995). Indeed, we calculated a higher SGD for larger tumours (SGD = 3.9) than for small tumours (SGD = 2.7). We reported a similar finding for the prodrug DNR-GA3 where the SGD increased in OVCAR-3 and MRI-H-207 xenografts when treatment of larger tumours was compared with that of small tumours (Houba et al, 1998). With respect to the clinical application, our findings are of interest in the context of the treatment of patients with large tumour deposits.

The glucuronide-prodrugs will probably not be activated in small tumours because they are non-necrotic and thus do not contain extracellular β-glucuronidase. In these tumours the prodrug may be used in combination with a second approach: Antibody-Directed Enzyme-Prodrug Therapy (ADEPT). In ADEPT, prodrugs are activated in tumours by an administered tumour-specific monoclonal antibody-enzyme conjugate. We have shown earlier that a conjugate of monoclonal antibody 323/A3 and human β-glucuronidase bound to tumour cells can activate anthracycline prodrugs in an efficient manner (Haisma et al, 1992). In addition to the chemically prepared conjugate a single chain Fv fusion protein may be used (Haisma et al, 1998). If such a tumour-specific conjugate is administered before DOX-GA3 injection, activation could also occur in the non-necrotic smaller tumour lesions such as micrometastases or minimal residual disease.

In conclusion, DOX-GA3 is a non-toxic prodrug which is specifically activated to DOX in tumour tissue by β-glucuronidase released by necrotic cells. Although the treatment schedule is not yet optimized we have already demonstrated that DOX-GA3 has a higher therapeutic index than DOX. These results are encouraging and warrant clinical development of DOX-GA3.

### ACKNOWLEDGEMENTS

This work was supported by the Dutch Cancer Society. We are grateful to CAM Erkelens for technical assistance.

### REFERENCES

Bosslet K, Czech J and Hoffmann D (1994) Tumor-selective prodrug activation by fusion protein-mediated catalysis. *Cancer Res* **54**: 2151–2159

Bosslet K, Czech J and Hoffmann D (1995) A novel one-step tumor-selective prodrug activation system. *Tumor Targeting* **1**: 45–50

Boven E, Winograd B, Fodstad O, Lobbezoo MW and Pinedo HM (1988) Preclinical phase II studies in human tumor lines: a European multicenter study. *Eur J Cancer Clin Oncol* **24**: 567–573

Boven E, Schluper HM, Erkelens CA and Pinedo HM (1990) Doxorubicin compared with related compounds in a nude mouse model for human ovarian cancer. *Eur J Cancer* **26**: 983–986

Boven E, Hendriks HR, Erkelens CA and Pinedo HM (1992) The anti-tumour effects of the prodrugs N-l-leucyl-doxorubicin and vinblastine-isoleucinate in human ovarian cancer xenografts. *Br J Cancer* **66**: 1044–1047

Connors TA and Whisson ME (1966) Cure of mice bearing advanced plasma cell tumours with aniline mustard: the relationship between glucuronidase activity and tumour sensitivity. *Nature* **210**: 866–867

de Jong J, Guerand WS, Schoofs PR, Bast A and van der Vijgh WJ (1991) Simple and sensitive quantification of anthracyclines in mouse atrial tissue using high-performance liquid chromatography and fluorescence detection. *J Chromatogr* **570**: 209–216

de Jong J, Geijssen GJ, Munniksma CN, Vermorken JB and van der Vijgh WJ (1992) Plasma pharmacokinetics and pharmacodynamics of a new prodrug N-l-leucyldoxorubicin and its metabolites in a phase I clinical trial. *J Clin Oncol* **10**: 1897–1906

Deprez-de Campeneere D, Baurain R and Trouet A (1982) Accumulation and metabolism of new anthracycline derivatives in the heart after IV injection into mice. *Cancer Chemother Pharmacol* **8**: 193–197

Eisenthal R and Cornish-Bowden A (1974) The direct linear plot. A new graphical procedure for estimating enzyme kinetic parameters. *Biochem J* **139**: 715–720

Fishman WH (1970) *Metabolic conjugation and metabolic hydrolysis.* Academic Press: New York, London

Haisma HJ, Boven E, van Muijen M, de Jong J, van der Vijgh WJ and Pinedo HM (1992) A monoclonal antibody-beta-glucuronidase conjugate as activator of the prodrug epirubicin-glucuronide for specific treatment of cancer. *Br J Cancer* **66**: 474–478

Haisma HJ, Sernee MF, Hooijberg E, Brakenhoff RH, v.d. Meulen-Muileman IH, Pinedo HM and Boven E (1998) Construction and characterization of a fusion protein of single-chain anti-CD20 antibody and human beta-glucuronidase for antibody-directed enzyme prodrug therapy. *Blood* **92**: 184–190

Hamilton TC, Young RC, McKoy WM, Grotzinger KR, Green JA, Chu EW, Whang-Peng J, Rogan AM, Green WR and Ozols RF (1983) Characterization of a human ovarian carcinoma cell line (NIH:OVCAR-3) with androgen and estrogen receptors. *Cancer Res* **43**: 5379–5389

Houba PH, Leenders RG, Boven E, Scheeren JW, Pinedo HM and Haisma HJ (1996) Characterization of novel anthracycline prodrugs activated by human beta-glucuronidase for use in antibody-directed enzyme prodrug therapy. *Biochem Pharmacol* **52**: 455–463

Houba PHJ, Boven E, Leenders RGG, Pinedo HM and Haisma HJ (1998) The efficacy of the anthracycline prodrug daunorubicin-GA3 in human ovarian cancer xenografts. *Br J Cancer* **78**: 1600–1606

Houba PH, Boven E, van der Meulen-Muileman IH, Leenders RG, Scheeren JW, Pinedo HM and Haisma HJ (1999) Distribution and pharmacokinetics of the prodrug daunorubicin-GA3 in nude mice bearing human ovarian cancer xenografts. *Biochem Pharmacol* **57**: 673–680

 © 2001 Cancer Research Campaign

Kearney AS (1996) Prodrugs and targeted drug delivery. *Adv Drug Deliv Rev* **19**: 225–239

Leenders RGG, Gerrits KAA, Ruijtenbeek R, Scheeren HW, Haisma HJ and Boven E (1995) β-Glucuronyl carbamate based pro-moieties designed for prodrugs in ADEPT. *Tetrahedron Lett* **36**: 1701–1704

Leenders RG, Damen EW, Bijsterveld EJ, Scheeren HW, Houba PH, van der Meulen-Muileman IH, Boven E and Haisma HJ (1999) Novel anthracycline-spacer-beta-glucuronide, -beta-glucoside, and -beta-galactoside prodrugs for application in selective chemotherapy. *Bioorg Med Chem* **7**: 1597–1610

Levvy GA and Conchie J (1966) β-glucuronidase and the hydrolysis of glucuronides. In: *Glucuronic acid, free and combined*, Dutton GJ (ed) pp. 301–364. Academic Press: New York

Martin GR and Jain RK (1994) Noninvasive measurement of interstitial pH profiles in normal and neoplastic tissue using fluorescence ratio imaging microscopy. *Cancer Res* **54**: 5670–5674

Molthoff CF, Calame JJ, Pinedo HM and Boven E (1991) Human ovarian cancer xenografts in nude mice: characterization and analysis of antigen expression. *Int J Cancer* **47**: 72–79

Schumacher U, Adam E, Zangemeister-Wittke U and Gossrau R (1996) Histochemistry of therapeutically relevant enzymes in human tumours transplanted into severe combined immunodeficient (SCID) mice: nitric oxide synthase-associated diaphorase, beta-D-glucuronidase and non-specific alkaline phosphatase. *Acta Histochem* **98**: 381–387

Sinhababu AK and Thakker DR (1996) Prodrugs of anticancer agents. *Adv Drug Deliv Rev* **19**: 241–273

Tomino S and Paigen K (1975) Purification and chemical properities of mouse liver lysosomal (L form) beta-glucuronidase. *J Biol Chem* **250**: 8503–8509

Weiss RB (1992) The anthracyclines: will we ever find a better doxorubicin? *Semin Oncol* **19**: 670–686

Young CW, Yagoda A, Bittar ES, Smith SW, Grabstald H and Whitmore W (1976) Therapeutic trial of aniline mustard in patients with advanced cancer. *Cancer* **38**: 1887–1895

Yuan F, Baxter LT and Jain RK (1991) Pharmacokinetic analysis of two-step approaches using bifunctional and enzyme-conjugated antibodies. *Cancer Res* **51**: 3119–3130

*© 2001 Cancer Research Campaign*
